# Conserving the Stage: Climate Change and the Geophysical Underpinnings of Species Diversity

**DOI:** 10.1371/journal.pone.0011554

**Published:** 2010-07-14

**Authors:** Mark G. Anderson, Charles E. Ferree

**Affiliations:** The Nature Conservancy, Boston, Massachusetts, United States of America; Duke University, United States of America

## Abstract

Conservationists have proposed methods for adapting to climate change that assume species distributions are primarily explained by climate variables. The key idea is to use the understanding of species-climate relationships to map corridors and to identify regions of faunal stability or high species turnover. An alternative approach is to adopt an evolutionary timescale and ask ultimately what factors control total diversity, so that over the long run the major drivers of total species richness can be protected. Within a single climatic region, the temperate area encompassing all of the Northeastern U.S. and Maritime Canada, we hypothesized that geologic factors may take precedence over climate in explaining diversity patterns. If geophysical diversity does drive regional diversity, then conserving geophysical settings may offer an approach to conservation that protects diversity under both current and future climates. Here we tested how well geology predicts the species diversity of 14 US states and three Canadian provinces, using a comprehensive new spatial dataset. Results of linear regressions of species diversity on all possible combinations of 23 geophysical and climatic variables indicated that four geophysical factors; the number of geological classes, latitude, elevation range and the amount of calcareous bedrock, predicted species diversity with certainty (*adj. R^2^ = 0.94*). To confirm the species-geology relationships we ran an independent test using 18,700 location points for 885 rare species and found that 40% of the species were restricted to a single geology. Moreover, each geology class supported 5–95 endemic species and chi-square tests confirmed that calcareous bedrock and extreme elevations had significantly more rare species than expected by chance (P<0.0001), strongly corroborating the regression model. Our results suggest that protecting geophysical settings will conserve the stage for current and future biodiversity and may be a robust alternative to species-level predictions.

## Introduction

As a result of climate change, conservation scientists have been developing a variety of methods for anticipating impacts and identifying priority places to protect in order to maintain biodiversity. The most commonly employed approaches are models that relate species ranges to habitats and climates, and then predict where species are likely to experience extreme turnover or have the highest stability [1]. The latter areas, being regions of low turnover, could be prioritized as refugia for the largest number of species. A second, often advocated approach is to simply provide an abundance of habitat corridors so that species can move around as their ranges shift [2]. Overall, many existing conservation plans simply don't account for changes in species distributions and clearly need revision. However, because land protection decisions are long term, resource intensive, and difficult to reverse, conservationists need a robust model for identifying reserve networks that is neither rendered obsolete by a changing climate, nor constantly in flux.

Here we explore a contrasting approach, which asserts that rather than trying to protect biodiversity one-species at a time, the key is to protect the ultimate drivers of biodiversity. The world has always experienced some measure of climate change and species ranges are not fixed. Accordingly, we should seek to maintain the landscape features that ultimately control species richness. A long-standing hypothesis in biogeography is that species richness is largely controlled by habitat heterogeneity [3], [4]. If this is true, then the best response to climate change might be the protection of a network of nature reserves that encompasses the maximum habitat heterogeneity [5], [6]. If, for example, geophysical diversity maintains species diversity, independent of climate, then conserving geophysical diversity may offer an approach to conservation that protects diversity under both current and future climates.

To test this hypothesis we used information on species richness, combined with a new comprehensive database of spatial data on geology, elevation, climatic averages and extremes, and over 18,705 rare species locations, to ask how much variation in species richness among 14 US states and three Canadian provinces is explained by geophysical factors.

We chose to focus on geology because geology defines the available environments, determines the location of key habitats, and stimulates diversification [7]. Although climate factors may drive diversity at continental scales, within a single climatic region like the temperate Northeast, geophysical factors may take precedence over climate in explaining diversity patterns [3], [8], and can overwhelm local biotic interactions [9]. In essence, geology directly shapes species diversity patterns through its influence on the chemical and physical properties of soil and water, and by creating topography that redistributes climatic effects creating predictable weather patterns and microclimates.

Evidence from the genetics of edaphic endemics suggests that the relationship between species and geology is not purely coincidental. New species may arise from sympatric populations of a parent species if presented with a novel environment. Consider the divergence of *Layia discoidea*, a serpentine endemic, from a more widely distributed species characteristic of sandy soils, [10], or the derivation of *Stellaria arenicola*, a boreal dune endemic, from the sympatric gene pool of a progenitor species (*S. longipes*) that lacks dune adaptive traits [11]. It appears that unusual or contrasting geologies can stimulate speciation even without strong barriers to gene flow, supporting the idea that discontinuous contrasting geologies may play a large role in evolutionary diversification [12].

The region we studied is dominated by rocky acidic soils derived from sandstones and granites. Years of inventory suggest that the region's rare and unusual biota is associated with areas of contrasting soils like those associated with fertile limestone, barren serpentine, or threatened coastal sands. Here, we were concerned with determining just how correlated species diversity is with geophysical diversity, and in measuring the magnitude of the latter's influence relative to climate. We hypothesized that if geophysical factors are an important driver of biodiversity then the diversity patterns of both common and rare species in eastern North America should be predictable from large scale geological patterns. Specifically we hypothesized that:

1. The total diversity of plants, vertebrates, and macro-invertebrates in each of 17 states and provinces is directly related to the number of contrasting geologic classes found within its boundaries.
2. When tested together, geophysical variables will supersede climatic variables in explaining regional biodiversity patterns.
3. Rare species will mostly be restricted to a single geologic setting and certain geological classes will show a consistently higher diversity of both rare and common species.

## Methods

### Study Area

**Figure 1:**
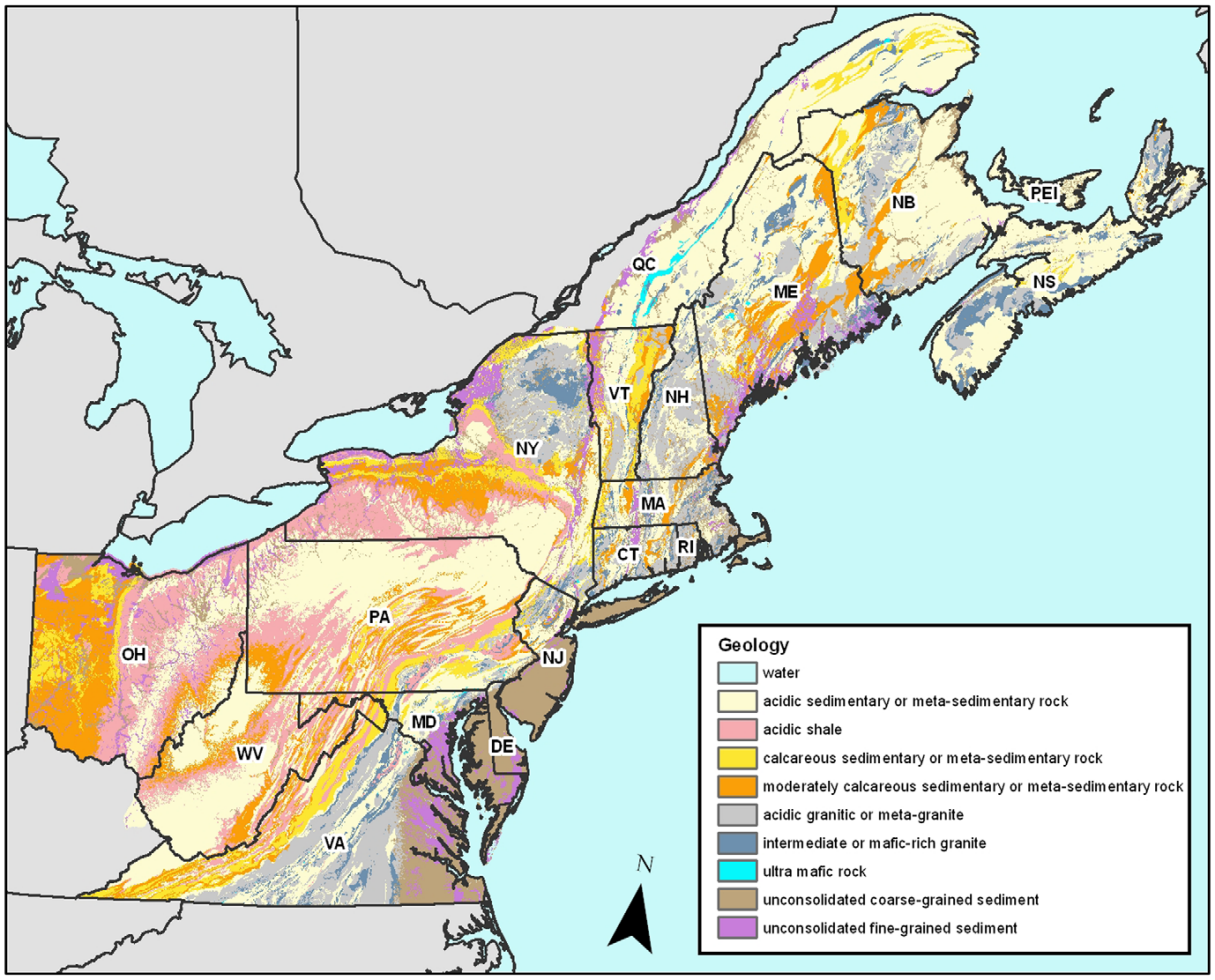
Map of the study region showing the geologic classes and state/province boundaries. Key to abbreviations: Maine (ME), New Hampshire (NH), Vermont (VT), New York (NY), Massachusetts (MA), Rhode Island (RI), Connecticut (CT), Pennsylvania (PA), Delaware (DE), New Jersey (NJ), Maryland (MD), Ohio (OH), West Virginia (WV), Virginia (VA), New Brunswick (NB), Nova Scotia (NS) and Prince Edward Island (PE).

The region studied covers 870,247 km^2^ (roughly twice the size of California), supports over 13,500 species of plants, vertebrates and macro-invertebrates, and has a wide diversity of lithologies and topography (Figure 1). The geographic area is defined by political boundaries corresponding to the New England and Mid Atlantic regions of the US and the Maritime Provinces of Canada. In all, the region includes seventeen states and provinces: Maine, New Hampshire, Vermont, New York, Massachusetts, Rhode Island, Connecticut, Pennsylvania, Delaware, New Jersey, Maryland, Ohio, West Virginia, Virginia, New Brunswick, Nova Scotia, and Prince Edward Island.

Our sample units were, by necessity, politically-defined, and do not follow ecological boundaries. However, these units had two important qualities that made them uniquely suitable for testing our hypotheses. First, the number of species present in them has been empirically determined based on decades of field inventory, combined with scientific literature, museum specimens, species lists, and reliable documented observations collected by local data centers [13]. Moreover, active Natural Heritage programs, or Conservation Data Centers, in every state or province maintain an inventory of all rare species locations within their boundaries. Second, the boundaries of each unit include a large variety of geology classes and elevation zones. Although the units differ greatly in size (2,822 km^2^ to 126,007 km^2^), the number and types of geological classes, the elevation range, and the latitude, are a function of the shape and location of unit with respect to regional geological patterns and are not correlated with the size of the unit (Figure 1., Pearson's correlation of area with: number of geology classes r = 0.32, P =  0.21, elevation range r = 0.43, P =  0.09, latitude r = −.0.15, P = 0.54).

### Data sets

#### Species

We tabulated the total number of species of vascular and non-vascular plant, vertebrates, and macro-invertebrates documented for each state and province [13]. We allowed for subspecies and varieties, noted whether each species was native or exotic, and excluded marine species. Additionally, we compiled 18,705 point locations of all rare species for which there was comprehensive inventory data across the region. These data included all species, subspecies, and varieties, that were ranked as critically imperiled (G1 or T1), imperiled (G2 or T2) or vulnerable (G3 or T3) by NatureServe [13] (hereafter referred to as “rare species”). The point locations were provided by US State Natural Heritage programs and by Maritime Canada's Conservation Data Center, and used with permission.

#### Geology class

We created a spatially comprehensive regional data base of geology classes at a resolution of 30 m by obtaining digital bedrock and surficial geology data layers from each state and province, and compiling the individual source maps into a single layer in digital form at the scale of 1:125,000 (Fig. 1). We grouped the 200+ bedrock types into nine lithogeochemical classes based on genesis, chemistry, weathering properties, and the textures of soils derived from the class. Our classification system expands on Robinson et al. [14], and is irrespective of age or degree of metamorphism. Seven classes were bedrock based and two were based on surficial deposits (Table 1).

**Table 1:** The geological classes and the lithologies included in each class.

Geology Class	Included Lithologies
**Ultramafic**: magnesium rich alkaline rock.	Serpentine, soapstone, pyroxenite, dunite, peridotite, talc schist
**Mafic**: quartz poor alkaline to slightly acidic rock.	Anorthosite, gabbro, diabase, basalt, diorite, andesite, syenite, trachyte, Metamorphic equivalents: Greenstone, amphibolites, epidiorite, granulite, bostonite, essexite
**Acidic Granitic**: quartz rich, acidic igneous and metamorphic rock.	Granite, granodiorite, rhyolite, felsite, pegmatite, Metamorphic equivalents: Granitic gneiss, charnocktites, migmatites
**Acidic Sedimentary**: fine to coarse grained, acidic sedimentary rock.	Mudstone, claystone, siltstone, Non-fissile shale, sandstone, breccia, conglomerate, greywacke, arenites, Metamorphic equivalents: slate, phyllite, pelite, schist, pelitic schist, granofel, quartzite
**Acidic Shale**: fine grained acidic sedimentary rock with fissile texture.	Fissile shale
**Calcareous Sedimentary**: Alkaline, soft sedimentary rock with high calcium content.	Limestone, dolomite, dolostone, other carbonate-rich clastic rocks, Metamorphic equivalents: Marble
**Moderately Calcareous Sedimentary**: Neutral sedimentary rock with some calcium.	Calcareous shale and sandstone, calc-silicate granofel, Metamorphic equivalents: calcareous schists and phyllite
**Fine Sediment**: fine-grained surficial sediments.	Unconsolidated mud, clay, drift, ancient lake deposits
**Coarse Sediment**: coarse-grained surficial sediments.	Unconsolidated sand, gravel, pebble, till.

The first eight are bedrock classes, and the last two are surficial classes.

#### Elevation

We compiled a regional elevation data layer directly from USGS 30 m digital elevation models. For the chi-square tests of rare species distributions, we defined six categorical elevation zones across the region based on dominant vegetation. While the six zones are well recognized, the exact boundaries between zones are highly variable across the region. Exploratory tests suggested that our results were not particularly sensitive to the exact choice of boundary thresholds, and thus we used a simplified scheme generalized from the distribution limits of dominant tree species [15]. The zones were variable in total area and included two small but distinct zones corresponding to coastal and alpine environments. The cutoffs used were: a) 0–6 m, coastal zone; b) 6–244 m, very low elevation, oaks, oak-pine, floodplain; c) 244–518 m, low elevation, hemlock-white pine-northern hardwoods, d) 518–762 m, mid elevation, northern hardwoods, spruce-hardwoods; e) 762–1097 m, high elevation, spruce-fir, f) greater than 1097 m, very high elevation, alpine and subalpine.

#### Climate

We used an existing climate dataset [16] to calculate seven climate variables for each state and province: 1) mean annual temperature, 2) mean diurnal temperature range, 3) mean annual temperature range, 4) mean annual precipitation, 5) precipitation during the warmest quarter, 6) minimum temperature during the coldest month, and 7) mean temperature during the coldest quarter. All were calculated from monthly means recorded over a 30 year period.

### Data Analysis

For each political unit, we calculated the total number of plants, vertebrates and macro-invertebrates present, and 23 auxiliary variables: the total number of geology classes and the area covered by each of the nine possible classes, the seven climate variables discussed above, the minimum elevation, maximum elevation and elevation range, the total area of the unit (area and ln area) and its central latitude. To determine the best fitting models, and the relative influence of each variable on diversity, we ran linear regressions of species richness per unit on all possible combinations of variables. We examined the AIC_c_ and R^2^ value for the best 1400 models, and considered the models with the highest adjusted R^2^ values and the lowest AIC_c_ values to be the best fitting models. We calculated the relative importance value of each variable by summing the AIC_w_ of all the models where the variable of interest was included [17]. For the latter calculations we used all models with a lower AIC_c_ value than the highest single variable model (415 models).

Finally, we plotted the results of the model with the highest R^2^ and lowest AIC_c_, and we examined the relationships revealed when this model was applied separately to plants, vertebrates and invertebrates to gauge its generality. Subsequently, we repeated the entire analytical procedure separately for each taxonomic group, for native species only, and for introduced species only, and we compared and summarized the results across the different groups.

Because the relationship between species diversity and area is so well established, we ran a second series of models to examine the influence of geophysical variables after factoring out variation explained by area. To do this, we ran a linear regression of all-species on the natural log of the area of each unit. Using the residuals from that model as a measure of the variance unexplained by area, we repeated the analysis described above, testing all possible variable combinations, to predict the residual variation in species diversity. We examined the 1400 best models representing all possible combinations of variables and considered the models with the highest adjusted R^2^ values and the lowest AIC_c_ values to be the best fitting.

To measure the relative restrictedness of rare species to a geology class or elevation zone, we overlaid (in a GIS environment) 18,705 rare species point locations on the geophysical spatial data and tabulated the geological class and elevation for each point intersection. Subsequently, for each species we calculated the number of classes, or zones, across which it was found. To determine if particular geology classes or elevation zones supported more rare species locations than would be expected by chance, we calculated the expected number of rare species locations for each category based on its proportional distribution in the region. We used a chi-square test to compare the observed distribution with the expected distribution. All data analysis was performed in JMP 8.

## Results

Results of running all possible linear combinations of the predictor variables identified 284 models that had R^2^ values greater than or equal to 0.90. Most of these models used four variables although it ranged from three to five. Across all models, the eight variables with the highest relative importance scores included: the number of geology classes (AIC_w_ = 0.99), calcareous bedrock (AIC_w_ = 0.83), latitude (AIC_w_ = 0.53), maximum elevation (AIC_w_ = 0.42), elevation range (AIC_w_ = 0.35), mean annual temperature (AIC_w_ = 0.20), average temperature of coldest month (AIC_w_ = 0.12) and acidic shale (AIC_w_ = 0.10). All others variables had scores below 0.10.

**Figure 2:**
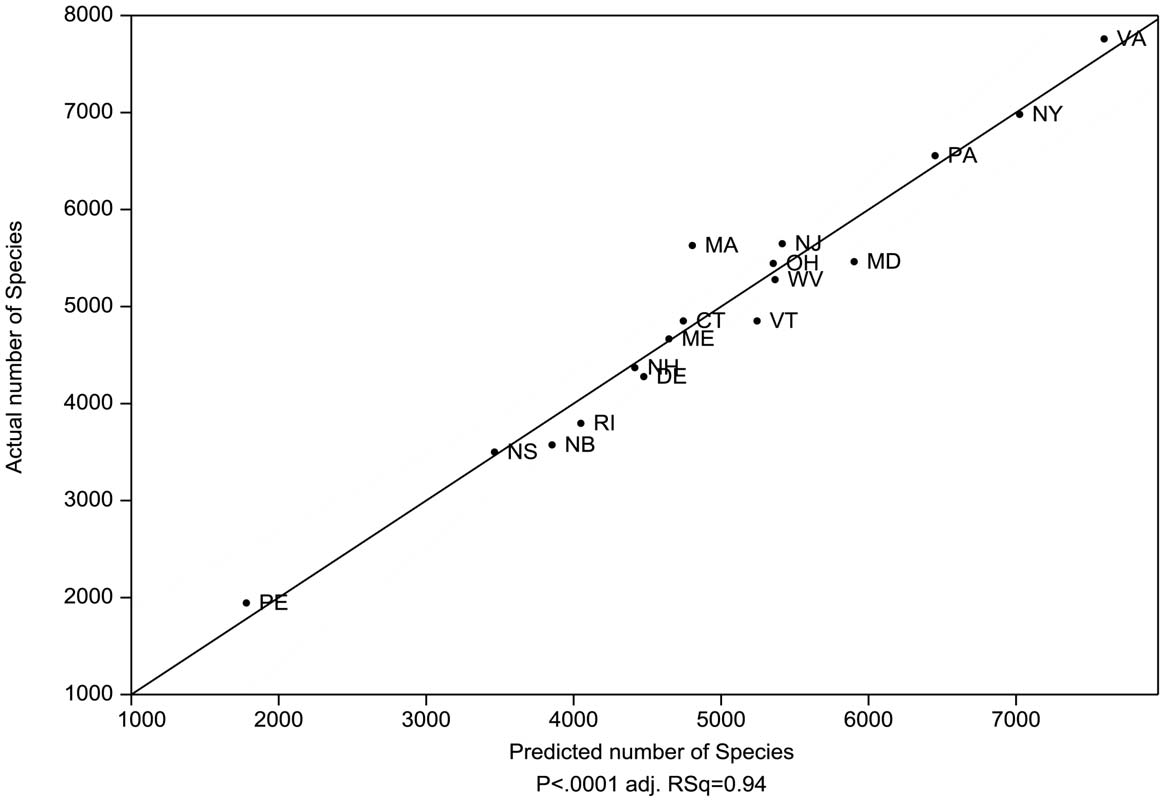
Actual species diversity plotted against the predicted diversity using the model with the highest R^2^ and lowest AIC_c_ (adj. R^2^ = 0.94, P<0.0001). This model is for all-species based on four factors. The model equation is Species Diversity  = 4205.77+417.62 * number of geology classes +0.0006* hectares of calcareous bedrock −0.0004*degree latitude +0.129* elevation range. Dashed line indicates 95% confidence interval. See Figure 1. for state and province legend.

The single model with the highest R^2^ and lowest AIC_c_ consisted of a four-variable linear regression that predicted species diversity with high certainty (adj. R^2^ = 0.94, P<0.0001, Figure 2). This model used four of the five most important variables: 1) the number of geology classes, 2) latitude, 3) elevation range, and 4) the amount of calcareous bedrock.

The ten models with the lowest AIC_c_ (<  = 264.0), all included the number of geology classes, and commonly calcareous bedrock, latitude, and max elevation (Table 2). Highly correlated variables; maximum elevation with elevation range (r  = 0.99), and mean annual temperature with latitude (r  = −0.92), substituted for each other in the top models, although latitude (0.52) was considerably higher in relative variable importance than mean annual temperature (0.20).

**Table 2:** The ten models with the highest R^2^ and lowest AIC_c_ arranged by AIC weights (AIC_w_).

UNADJUSTED MODEL: Variables in the ten best models											
# Variables	**4**	**4**	**4**	**4**	**3**	**4**	**4**	**4**	**4**	**5**	
R^2^	0.96	0.96	0.96	0.96	0.94	0.95	0.95	0.95	0.95	0.96	
AIC_c_	260.6	260.6	260.6	261.2	262.2	263.0	263.7	263.8	263.9	263.9	
AIC_w_	0.192	0.189	0.187	0.140	0.084	0.057	0.040	0.038	0.037	0.037	**T**
# Geo class	x	x	x	x	x	X	x	x	x	x	**10**
Calcareous	x	x	x	x	x	X		x	x	x	**9**
Latitude	x		x		x		x				**4**
Max Elev.		x	x			X				x	**4**
Elev. Range	x			x				x			**3**
Mean Temp		x		x						x	**3**
Ave of Coldest						X		x	x		**3**
Acid shale							x			x	**2**
Min of coldest									x		**1**
Granite							x				**1**

RESIDUAL MODEL (area adjusted): Variables in the ten best models											
# Variables	**3**	**4**	**4**	**4**	**4**	**4**	**4**	**4**	**4**	**3**	
R^2^	0.84	0.88	0.87	0.87	0.87	0.86	0.86	0.86	0.86	0.81	
AIC_c_	270.4	270.6	271.7	272.0	272.6	272.8	273.0	273.1	273.1	273.4	
AIC_w_	0.219	0.198	0.114	0.101	0.074	0.068	0.061	0.058	0.058	0.050	**T**
# Geo class	x	X	x	x	x	X	x	x	x	x	**10**
Acid sed.	x	X	x	x	x	X	x		x		**8**
Latitude	x	X	x		x					x	**6**
Calcareous		X		x		X					**3**
Acid shale					x		x		x		**3**
Mean Temp				x			x				**2**
Ave of Coldest						X			x		**2**
Precip warm 1/4			x							x	**2**
Min of coldest								x			**1**
Granite								x			**1**
Mafic								x			**1**

The unadjusted model included area as a possible variable. In the residual model, species diversity explained by area was first factored out of the model.

Regressing the total number of species singly on unit area (ln hectare) indicated a moderate positive relationship (R^2^ = 0.346, P  = 0.01) between area and diversity. Results for the models based on residuals from this species-area regression were similar to the results from the unadjusted model; however no model using the residuals had an R^2^ over 0.90. In all, 105 models had an R^2^ greater than or equal to 0.80. Variables with consistently high relative importance scores included: the number of geology classes (AIC_w_ = 0.89), the amount acidic sedimentary bedrock (AIC_w_ = 0.52), latitude (AIC_w_ = 0.50), mean annual temperature (AIC_w_ = 0.21), the amount of calcareous bedrock (AIC_w_ = 0.15), and the average temperature of the coldest month (AIC_w_ = 0.15). The ten models with the lowest AIC_c_ (< =  173.0) all included the number of geology classes (Table 2).

**Figure 3:**
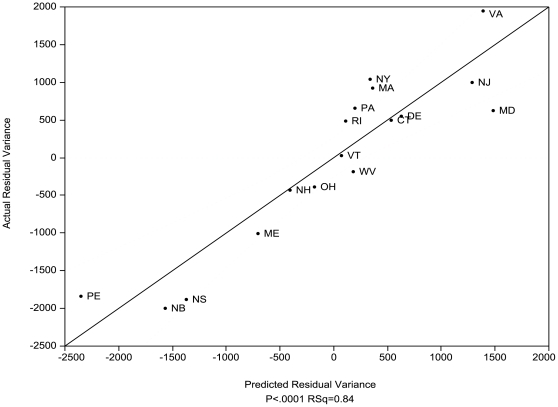
Residual variance in species diversity after area was factored out. The figure shows the actual species numbers plotted against the predicted variance for the model with the highest R^2^ and lowest AIC_c_ (adj. R^2^ = 0.84, P<0.0001) for all species based on three factors: number of geology types, latitude and the amount of acidic sedimentary bedrock (negative). Dashed line indicates 95% confidence interval. See Figure 1. for state and province legend.

The single linear regression with the highest adj. R^2^ and AIC_w_ for the residuals included the three most important variables: the number of geology classes, latitude and the amount of acidic sedimentary bedrock relationship (adj. R^2^ = 0.84, P<0.01, Figure 3). In contrast to the other variables, acidic sedimentary bedrock had a negative relationship to diversity.

**Figure 4:**
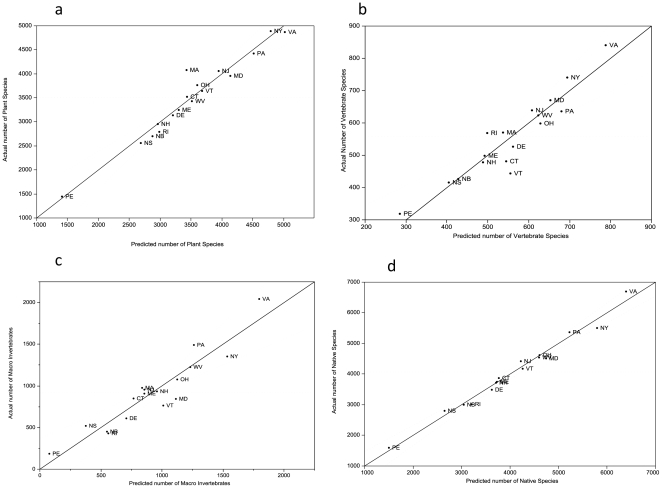
The all-species model using number of geology classes, latitude, amount of calcareous bedrock and elevation range. Applied to plants only (a), vertebrates only (b), invertebrates only (c) and native species only (d.). All models have P<0.0001; dashed line indicates 95% confidence interval. See Figure 1 for state and province legend.

Examination of the individual taxonomic groups indicated that the all-species model based on geology classes, latitude, calcareous bedrock and elevation range, performed well when applied to the separate taxa: plants (adj. R^2^ = 0.95, Figure 4a), vertebrates (adj. R^2^ = 0.87, Figure 4b), and macro-invertebrates (adj. R^2^ = 0.88, Figure 4c). The individual best–fit models for the taxonomic groups differed slightly from the model based on all-species (Table 3); however many of the best fitting models had similar AIC_c_ values. Running the model separately for native species (R^2^ = 0.975, Figure 4d) and introduced species (R^2^ =  0.913) gave comparable results to the all-species models, although in the model for introduced species, acidic shale was a stronger predictor than calcareous bedrock.

**Table 3:** Comparison of models with highest R^2^ and lowest AIC_c_ for individual taxonomic groups.

Variables	All Species	Plants	Vertebrates	Invertebrates	Natives
# of geology classes	*0.0004*	*<0.0001*	*0.0419*		*0.0005*
Amt of calcareous bedrock	*<0.0001*	*<0.0001*		*0.0034*	*<0.0001*
Amt of coarse sediments			*0.0005*		
Elevation range	*0.0343*			*0.0009*	*0.0004*
Latitude	*0.0001*	*0.0019*	*0.0008*	*0.0003*	*<0.0001*
R^2^	0.956	0.945	0.933	0.8739	0.982
Adj R^2^	0.942	0.933	0.911	0.8448	0.975
P of Model	*<0.0001*	*<0.0001*	*<0.0001*	*<0.0001*	*<0.0001*
AIC_c_	207.37	191.24	133.16	182.59	177.97

The variable significance is given for all species, individual taxonomic groups and for native species only. Columns show the P value for each variable. For each model we give the R^2^, P-value and Akaike's second-order Information Criterion (AIC_c_, Burnham and Anderson 2002^11^).

**Figure 5:**
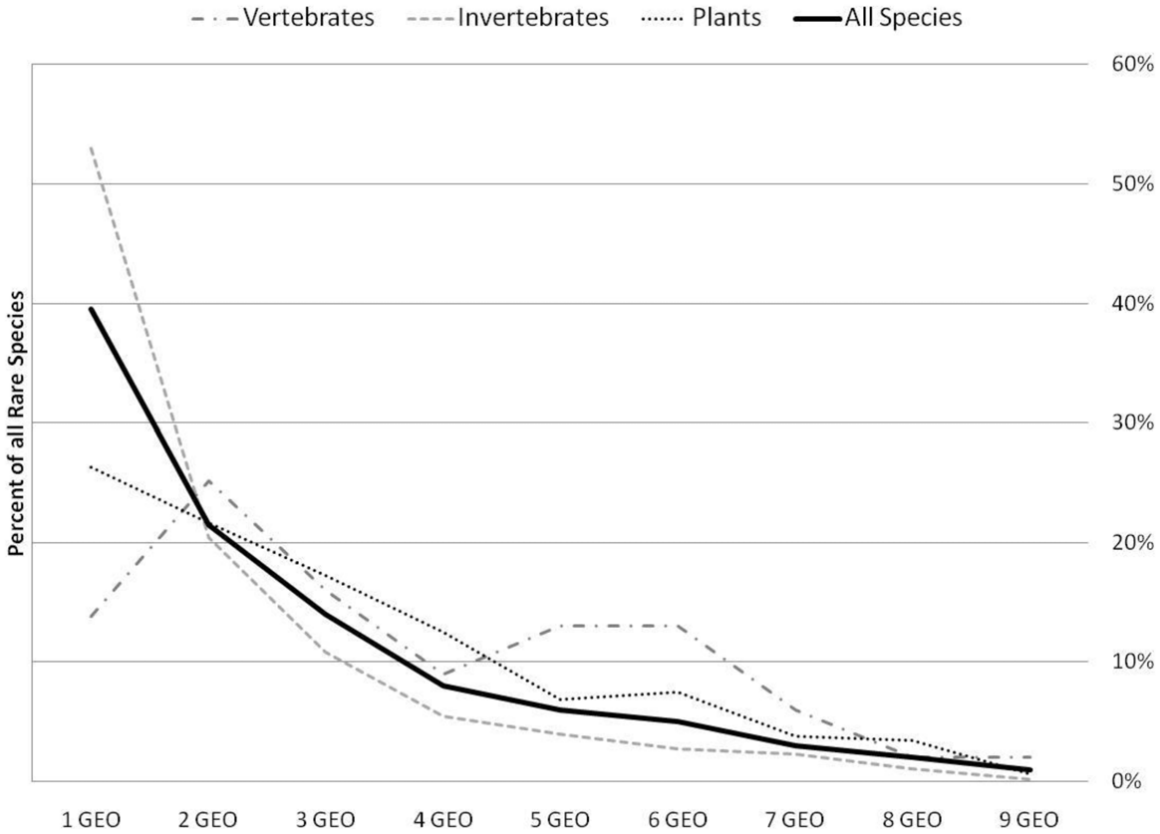
The proportion of each rare species group restricted to single or multiple geology classes. The x axis shows the number of geology classes and the y axis gives the proportion of the total rare species found across that many classes.

Overlay of the rare species location points on the geophysical spatial data revealed that 40% of the 885 rare species were restricted to a single geology class and another 21% were restricted to two (usually related) geology classes. Invertebrates were the most restricted group −53% fell on a single geology class - followed by plants (26%) and vertebrates (14%, Figure 5). Amphibians and fish were the most restricted vertebrate groups, and birds were the least restricted.

The number of restricted rare species ranged from five on ultramafic geology to 95 on moderately calcareous geology. Comparing the expected number of rare species per geology class (based on area) with the observed number, confirmed significant differences among the geology classes (P<0.0001, chi-square), and the elevation zones (P<0.0001, chi-square). Acidic sedimentary areas had significantly less rare species locations (−3096) than expected given its abundance in the region. Conversely, calcareous bedrocks (+605), coarse sediment (+2463), and fine surficial sediment (+337) all had higher densities of rare species locations than expected. Ultramafic bedrocks had a higher than expected density of rare plant locations. The results for the elevation zones indicated that extreme low (+1064) and extreme high elevations (+424) had the highest densities of rare species.

The distribution patterns of the rare species locations strongly corroborated the results of the regression models. First, each geology class contained a unique set of restricted species that were apparently endemic to the class. Second, as in the regression models, calcareous and coarse surficial geologies had the highest number of rare species locations across all taxonomic groups. Third, the elevation extremes had significantly more restricted species than intermediate elevations. Thus, based on rare species alone, having many geology classes and a wide elevation range appears to increase a state's chances of supporting specific rare species; having calcareous bedrock or coarse sediment increases those chances further.

## Discussion

Our results suggest that geological diversity, elevation range and latitude explain regional species diversity patterns within eastern temperate North American. The strong relationships we uncovered held for native and introduced species, for all taxonomic groups, and were present in models that both included or excluded area. In all models tested, geophysical variables had a larger influence, and were selected more often, than climatic variables. Further, rare species populations, comprising about 6% of the total flora and fauna, were largely restricted to a single geology class or elevation zone, and this may account for some of the species diversity associated with each geophysical setting. It is likely that while climatic factors drive diversity patterns at continental scales [18], [19], and defines the range of most species, geophysical factors determines where, within their range, the populations are located. Our evidence suggests that although the individual ranges of many species may shift with climate change, the spatial pattern of total biodiversity will remain associated with the enduring geophysical properties of the land.

The 13,500 species included in this region differ widely from north to south; the southernmost state (Virginia) shares only 30% of its biota with the northernmost province (New Brunswick), and the type of rare species associated with each geology class also differs geographically. Moreover, the region itself has been in flux for over a century, with many documented range shifts and extinctions, and over a third of the flora classified as introduced [13]. Given these historical patterns, our results suggest that distribution shifts, range expansions and contractions, or new species pools, do not undermine the basic relationships between species and geophysical factors. Thus, as we head into a period of dramatic climate-driven rearrangement of species distribution patterns, we assert that conserving a full spectrum different geology classes stratified across elevation zones and latitudes, may offer an approach to conservation that protects diversity under both current and future climates.

We had data for only one temperate region, but the importance of specific geophysical environments in harboring unique flora and fauna has been documented for a wide range of climatic regions [7]. Limestone glades, coastal dunes, serpentine pavements, basalt ridges, shale barrens and alpine summits are already the focus of conservation on several continents in a variety of climates. This is likely because the chemical and structural properties of soil and water are so fundamental to most species. For example, streams buffered by dissolved limestone (CaCO3) are more hospitable to acid-sensitive amphibian larva and richer in faunal diversity [20]. Many freshwater mussels, for instance, depend directly on calcium for shell growth and are consequently found in greatest abundance and diversity in streams that flow through limestone. Terrestrial limestone grasslands similarly carry a much richer flora and fauna than do acidic grasslands [21], while in contrast, serpentine soils are toxic to many species and those that thrive in them are often strict endemics with traits that enhance the tolerance of this condition.

Species are also sensitive to the physical structure of soils and landforms. For instance, species adapted to sand dunes exhibit traits to tolerate continuous burial. Endemics of sparsely vegetated “shale barrens,” are adapted to the constant downward movement of fissile shale plates on hot south-facing slopes [22]. Unique cave and karst features that form in limestone regions host of unique set of species found only on these landforms.

Rosenzweig [3] suggested that species richness is determined by environmental heterogeneity at scales below 10,000 km^2^, but the correlations in this study indicate that geophysical heterogeneity remains, or increases, in importance at greater scales. A similar pattern has been recognized in the southeastern US, where a disproportionate number of species persist relative to the area of the habitats, due to species narrowly restricted to specific habitats such as rocky outcrops, and a high level of endemism attributed to geologic history [23].

At the scale of this study, geologic heterogeneity was decidedly more important than area in explaining diversity patterns. Factoring out area in the regression model had little effect on the importance of key variables, and most of them had similar Akaike weights in the original and the area-corrected models: number of geology classes (AIC_w_ = 0.99 vs. 0.89), latitude (AIC_w_ = 0.53 vs. 0.50), mean annual temperature (AIC_w_ = 0.20 vs. 0.21), and average temperature of coldest month (AIC_w_ = 0.12 vs. 0.15). The relative importance of alkaline versus acidic bedrock, however, shifted notably. The strong positive relationship between diversity and calcareous bedrock in the original model was replaced by the negative relationship with acidic sedimentary bedrock in the area-corrected model. The opposing relationship that these two bedrock classes had with diversity was also seen in the rare species tests, suggesting that the diversity of the unit may be a factor of its overall acidity, measured either by the dominance of acidic bedrock or the mitigating presence of calcareous bedrock.

Visually, the influence of geological diversity can been seen by comparing, for example, Maryland, Vermont, and New Hampshire; three states that have almost equal areas but differ greatly in species diversity and in their corresponding geologic heterogeneity (Figure 1). Thus, our results add to the growing evidence that the influence of geophysical heterogeneity may override the species-area curve when the heterogeneity is not correlated with area [24].

The patterns we documented may not have emerged until the observation scale was broad enough to encompass several major geological formations and thus the results are relevant to conservation planning at large (ecoregional and regional) scales. At finer scales, such as within a single geologic class, the influence of topographic features may be the most important physical determinant of diversity [4], [6], [25], although occupancy of a fine scale feature by a rare species is difficult to predict [26].

The importance of geological heterogeneity may be widespread, but the influence of particular bedrock types is probably regionally dependent. In our study area, calcium rich limestone support rare bats, endemic cave invertebrates and an array of uncommon herbs. Although, a specific biota for limestone and karst has been documented on six of the seven continents [7], the influence of calcareousness on plant richness appears to depend on the substrate on which the regional flora evolved [27]. Many rare species were also associated with coarse sandy substrates. Further, the amount of coarse sediment was correlated with total species diversity (Pearson's r = 0.70) and was selected as a variable in the regression models for vertebrates and invertebrates (Table 3), suggesting that this is also an unusually important substrate in this region.

We used rare species location to corroborate the patterns found in the regression models based on all species, but most rare species have small and confined distributions, and the pattern of restriction we detected could be caused by chance as well as by physiological or ecological mechanisms. One robust conclusion we can draw from the results is that the relationships between species and certain geological classes - strongly positive for calcareous bedrock and somewhat negative for acidic sedimentary rock - were independently apparent in the distribution patterns of both common and rare species, and therefore probably not a spurious correlation. Importantly, we did not test whether the rare species distributions were confined to a limited climate space, although it is almost certain that most would show small climate envelopes reflecting their constrained ranges. A species restricted by both climate and geology could face a severe extinction risk particularly in areas that lack local connectivity.

We refer to latitude as a geophysical variable because it is a position metric tied to a specific geographic location on the earth's surface. The strong correlation between diversity and latitude has puzzled scientists for years, with over 25 mechanisms proposed to explain latitudinal diversity gradients, and no single one proving sufficient to explain the phenomena [28]. In this region, latitude correlates with temperature, day length, solar radiation, and the limits of historic glaciations, so it is likely a surrogate for a complex set of factors. Notably, latitude was a much more important variable in our models than temperature or any single climate factor correlated with it. From the point of view of conservation, stratifying a network of reserves across latitudinal gradients make good sense as a strategy for conserving diversity, because the latitude-diversity relationship holds under a variety of climates.

Current recommendations for addressing climate change in conservation planning largely focus on predicting future habitat for individual species based on climatic envelope models [2]. Although these models have helped catalyze attention, and clarify the thinking about climate change effects, their utility has come under question because they are hindered by large uncertainties, and often unrealistic assumptions. For example, many models assume that temperature alone sets range limits at both high and low extremes [29], that the realized niche is equivalent to the fundamental niche [30], and fail to account for biogeographic factors such as persistence and spread from isolated refugia [31]. From the perspective of our results, adding geology, elevation, and landforms to the models might allow for more realistic results and in many cases narrower predictions of suitable habitat.

The alternative approach of basing conservation on geophysical settings, rather than predicted distributions of individual species, may be more effective in conserving biodiversity over long time scales. We use the term “geophysical settings” instead of “geophysical heterogeneity” to emphasize that simply adding a small outcrop of an unusual geology to a conservation plan is unlikely contribute the full biota associated with that geology. Rather, we suggest that in each geophysical setting, conservationists will need to maintain a functioning ecosystem that allows for processes and dynamics, including species turnover. For instance, a geophysical setting target such as “low elevation forest on limestone” would need to be conserved at a large enough scale to sustain associated species populations and recover from disturbances [32], even as the composition changes through time.

Instead of aiming to maintain a specific composition, the in-situ conservation of ecosystems defined by geophysical settings puts more emphasis on accommodating dynamic processes, maintaining ecological function and building adaptive capacity. Analogous to Hutchinson's [33] “ecological theaters,” protecting geophysical settings is a way of conserving the stage for future communities characterized by a biota specific to the site conditions. Similarly, Rouget [34] argued that conservation should focus on the spatial components of ecological and evolutionary processes, referring in this case to the shared drivers of climate, geology and topography. These new approaches allow for species distributions to shift, and for novel communities to form, while still conserving the maximum biodiversity.

We emphasize that conserving geophysical settings is a strategy for long term conservation success in a dynamic climate, and not for preventing immediate local extinctions. Many of the climate envelope approaches are about getting specific species through the next 100 years, and if one designed conservation priorities solely around geology, we might still lose many species in the short term, particularly those with ranges confined by both geophysical and climatic factors. However, we expect that under rapid climate change scenarios there will be inevitable tradeoffs between efforts to conserve individual species, and conserving the environments that those species evolved in and are adapted to. Too much emphasis on individual species could distract from the broader, and more fundamental, loss of whole ecological settings.

Geophysical setting and individual species approaches are not incompatible. In addition to differences in temporal scale discussed above there is also a difference in geographic scale, with climate as the arbiter of broad patterns and geology the proximate factor defining the specific location of most species. Because species locations are so intertwined with geophysical properties, many current conservation areas chosen for a single population of a rare species, an unusual community type, or a taxonomic hotspot, already represent unique combinations of geophysical factors (e.g. serpentine barren or limestone fen) that benefit many species. In these cases it is a matter of redefining the conservation goals of the site to encompass a functioning ecosystem representing the specific geophysical setting. In rethinking the conservation of a site more attention will need to be paid to the scale and context of the protection, as our results increase the importance of connectivity. For instance, it is unrealistic to expect the species comprising a limestone valley bottom ecosystem to simply move up on to granite slopes, and thus it is necessary to consider how to prevent their isolation from other limestone settings, and how to maintain the flow of processes and species between like settings.
